# Increment in Dietary Potassium Predicts Weight Loss in the Treatment of the Metabolic Syndrome

**DOI:** 10.3390/nu11061256

**Published:** 2019-06-02

**Authors:** Brurya Tal, Jessica Sack, Marianna Yaron, Gabi Shefer, Assaf Buch, Limor Ben Haim, Yonit Marcus, Galina Shenkerman, Yael Sofer, Lili Shefer, Miri Margaliot, Naftali Stern

**Affiliations:** Thhe Sagol Center for Epigenetics of Aging and Metabolism, the Institute of Endocrinology, Metabolism and Hypertension, Tel Aviv-Sourasky Medical Center; Sackler Faculty of Medicine, Tel Aviv University, 6423906 Tel Aviv, Israel; bruryat@tlvmc.gov.il (B.T.); jessicasack8@gmail.com (J.S.); yaron5@orange.net.il (M.Y.); gabish@tlvmc.gov.il (G.S.); buchasaf@gmail.com (A.B.); lbenhaim1@gmail.com (L.B.H.); yonitm@tlvmc.gov.il (Y.M.); galina.shenkerman@gmail.com (G.S.); yaelso@tlvmc.gov.il (Y.S.); gabi.shefer@gmail.com (L.S.); mirim@tlvmc.gov.il (M.M.)

**Keywords:** dietary potassium, metabolic syndrome, weight loss

## Abstract

Background: In the treatment of obesity/metabolic syndrome, dietary measures traditionally focus on reducing carbohydrate/fat-related caloric intake. The possibility that changes in potassium consumption may be related to the achieved weight loss has not been previously explored. Methods: Sixty-eight participants, with a mean age of 51.6 ± 11.0 years (F/M—30/38), who fulfilled the ATPIII criteria for the metabolic syndrome (MS) were enrolled into a 1-year intensive multidisciplinary program. Nutritional recommendation consisted of a moderate low calorie/high protein Mediterranean diet. Baseline assessment included clinical and biochemical profiling, and body composition. Nutritional components were registered over 7 days before and at the end of 1 year of treatment. Results: Mean baseline body mass index (BMI) was 35 ± 4 kg/m², which declined by 9.4 ± 0.1% after one year of combined intervention. Linear stepwise regression analysis revealed that 45% of the predicted variance of the % decline in BMI was related to increased consumption of dietary potassium (β = −0.865) and caproic acid (β = −0.423) and reduction in the consumption of dietary vitamin B6 (β = 0.542), calcium (β = 0.335), total carbohydrates (β = 0.239) and total caloric intake (β = 0.238; *p* < 0.001). Notably, the strongest correlate of the decline in BMI was the increase in dietary potassium intake (β = −0.865). Subjects whose achieved decrease in BMI was above the average (*n* = 30) increased potassium intake by 25% as compared to an increase in dietary potassium intake of only 3% by those whose decline in BMI was below the average (*n* = 36; *p* < 0.05). The change in dietary potassium was related to the percent increase in dietary protein (r = 0.433; *p* < 0.001). Conclusion: An increase in dietary potassium consumption is a previously unrecognized predictor of the achieved reduction in BMI in a weight-loss-oriented multidisciplinary intervention in obesity/MS. Prospective trials are underway to confirm this post-hoc finding.

## 1. Introduction

In the treatment of obesity and the metabolic syndrome (MS), dietary measures focus on reducing carbohydrate/fat-related caloric intake. Less attention has been given to the possibility that other dietary components could be related to the achieved reduction in body mass index (BMI) during caloric restriction. Variation in electrolytes and mineral intake appears inherent to the choice of any particular dietary protocol applied in an attempt to promote weight loss. Further, large inter-subject variations in the implementation and compliance with the same diet are also inevitable in any weight loss program that utilizes a presumably uniform dietary approach but does not offer fixed/supplied, pre-made meals. Hence, subjects participating in the same weight loss program vary in the actual intake of electrolytes, minerals and other components.

Dietary consumption of potassium in the general population in Western countries appears to be substantially lower than the Dietary Recommended Intake (DRI) of ≥4.7 g. For example, in the National Health and Nutrition Examination Survey (NHANES) III, the average daily potassium intake in adults was 2.9–3.2 g for men and 2.1–2.3 g for women. [1,2,3,4]. Particularly impressive was the finding that only 10% of men and less than 1% of women consumed the DRI of potassium [2].

Two widely recommended diets entail an increase in the consumption of potassium: the Mediterranean diet and the DASH diet. In one study of the Mediterranean diet, better compliance with the diet was linked to higher consumption of dietary potassium as well as calcium and magnesium [5]. A greater than average consumption in potassium (around the 75th percentile of the US consumption) is also an inherent feature of the DASH diet [6,7]. Whereas many clinical trials showed that high potassium intake, such as provided by the DASH diet is linked to blood pressure reduction [8,9], the potential role of potassium intake in weight loss has thus far generated little interest. In the present report, we examined the potential association between the change in dietary intake of potassium and the achieved change in BMI in a cohort of subjects with the MS who participated in year-long multidisciplinary intervention program with a nutritional focus on the Mediterranean diet.

## 2. Methods

### 2.1. Study Design and Population

This report represents a post-hoc analysis of nutritional data from an ongoing study of intensive multidisciplinary year-long intervention in subjects with the MS, as described in detail in a previous publication [10]. The study was approved by the institutional review board of TASMC and was registered at the NIH (NCT03558685). Written informed consent was obtained from the participants. The entry period was 2010 to 2018. In the present report, we analyzed data from the first 68 consecutive recruited patients (F/M—30/38) who completed 1 full year of intervention. Subjects aged 18–70 were recruited through local advertisement provided that they fulfilled the diagnostic criteria for the MS as defined by the Third Report of the Adult Treatment Panel (ATPIII) [11]. Impaired fasting glucose was considered a glucose level ≥100 mg/dL but subjects with diabetes were not included. Additional exclusion criteria were the presence of current or recent pregnancy, intention to conceive within the trial’s period, chronic renal or liver disease and past bariatric surgery or current participation in any dietary/medical program with current continuous weight loss.

### 2.2. Study Outcomes

Baseline and end of study assessments included medical history, physical examination and biochemical profiling, region-defined body composition with dual-energy X-ray absorptiometry (Lunar iDXA; GE Healthcare, Wauwatosa, WI, USA) and the determination of resting metabolic rate (RMR) by indirect calorimetry (Cosmed Quark RMR; Rome, Italy). Plasma asymmetric dimethyl arginine (ADMA) and arginine were measured by HPLC with UV detection, following derivatization, as previously described in detail [12].

### 2.3. Study and Nutritional Intervention

Concomitant interventions included lifestyle modification with a personally tailored program of diet, as detailed below, and physical activity adjusted for age and specific physical limitations, targeting engagement in physical activity of at least 150 min/week. Lipid lowering and or blood pressure lowering drugs were prescribed as needed according to guideline-assisted medical practice. Patients were seen by a physician every 3 months. The dietitian had a weekly meeting with the patients for the first three months, every other week during months 4–6, once a month during months 7–9 and every 6 weeks during the last three months of the study.

Nutritional recommendation consisted of moderate caloric restriction, set at 25% to 30% less than calories needed for resting metabolic rate. We applied a high protein Mediterranean diet with the following food group distribution: 30% as protein (>0.8 g/kg/d); 40% as carbohydrates with medium/low glycemic index; 30% as fat (≥10% monounsaturated fatty acid, ≤7% saturated fatty acid, no trans fats, and 1.6 g omega-3 for men and 1.1 g omega-3 for women). Diet was rich in olive oil, fish, chicken, nuts, white milk products, fruits, and vegetables but low in artificial sugars, commercial sweets, pastries, butter, margarine, and red meat. Dietary fiber content ≥25 g/day [10].

### 2.4. Nutritional Assessment

Nutritional components were registered over 7 days before and at the end of the 1-year treatment, by detailed questionnaires subsequently entered into the nutritional analysis software program “Zameret”, developed by the Food and Nutrition Administration, Israel Ministry of Health (Version 2, 2007, Jerusalem, Israel). The questionnaire was completed by the participants and then reviewed by a dietician in the presence of the patient to resolve unclear points. In the first and final interview, subjects were asked to submit a 7-day diet record (24 h × 7) preceding the interview. During interviews conducted both at the beginning and conclusion of the 1-year study period, the interviewing dietician used the “Food and Food Quantity Guide” and measuring aids such as a tablespoon and a teaspoon, in an attempt to facilitate quantification of foods consumed.

### 2.5. Quality Control for Nutritional Assessment

After the input of the food consumption data was completed and transferred to the Excel format, the report data were examined for abnormal data, inappropriate quantities, discrepancies between hours and meals, missing quantities, and misuse of codes. All typing errors found were corrected.

### 2.6. Statistical Analysis

Data analysis was performed with SPSS software (24.0; IBM International, Armonk, NY, USA). All variables presented in this study are continuous and presented as mean ± SD. To examine the differences between subjects before and after the intervention, a *t*-test for paired samples was performed (see Table 1 and Table 2). The BMI change after one year was predicted using a multiple linear regression in a stepwise manner. The assessed nutrients after one-year of intervention in the linear model included the following (percentage of intake changes during the study): (1) macronutrients including carbohydrates (including dietary fiber and total sugars), proteins and fat (including mono-saturated fatty acids, poly-saturated fatty acids, cholesterol, as well as, specific fatty acids such as butyric, caproic, caprylic, capric, lauric, myristic, palmitic, stearic, oleic, linoleic, linolenic, arachidonic, docosahexanoic, palmitoleic, gadoleic, eicosapentaenoic, erucic); (2) micronutrients including calcium, iron, magnesium, phosphorus, potassium, sodium, zinc, copper, vitamin A, carotene, vitamin E, vitamin C, thiamin, riboflavin, niacin, vitamin B6, folate and vitamin B12. Pearson correlations were used to test specific correlations of change in potassium consumption with the change in the consumption of major food groups and components. We next conducted paired sample *t*-tests in order to examine the change in potassium intake within different kinds of foods. Also, we compared participants whose achieved change in BMI was below vs. above the group’s average change during the year of the study using an independent *t*-test. Finally, a repeated measures ANOVA was performed to examine the relation between the change in the potassium density (mg/Kcal/day) and the achieved change in BMI (above or under the average loss).

## 3. Results

### 3.1. Participants

The clinical and biochemical characteristics study cohort at the baseline as well as one year later, at the completion of the intervention, are summarized in Table 1. Diet-related features at the onset of the study and one year later are shown in Table 2. Mean age at the baseline was 52 ± 12.5 years with a mean BMI of 35 ± 4 kg/m² and % fat body mass (FBM) of 42 ± 7%. Initial daily energy consumption by dietary questionnaire was of 2999 ± 1071 Kcal/d and RMR was 1831 ± 403 Kcal/day.

Within the 1 year, participants lost, on average, 9.36 kg, translating into 3.29 ± 2.51 BMI units (*p* < 0.001), which represents a 9.4% ± 7% reduction in BMI. Multiple linear regression in stepwise manner analysis revealed that 45.6% of the predicted variance of the % change in BMI was related to the change (in %) in the consumption of potassium, vitamin B6, caporic acid, calcium, sugars consumption and total energy consumption. As can be seen in Table 3, the increase in potassium consumption was the strongest contributor to the prediction of the reduction in BMI (β = −0.865). Of note is also the association between the increases in the consumption of caporic acid at the end of the study, compared to the baseline recording, to BMI lost. Additionally, reduction in the consumption of vitamin B6, calcium, food energy and total sugars by the end of the intervention relative to the baseline consumption of these components at the initiation of the program was linked to a larger reduction in BMI (Table 3).

**Figure 1:**
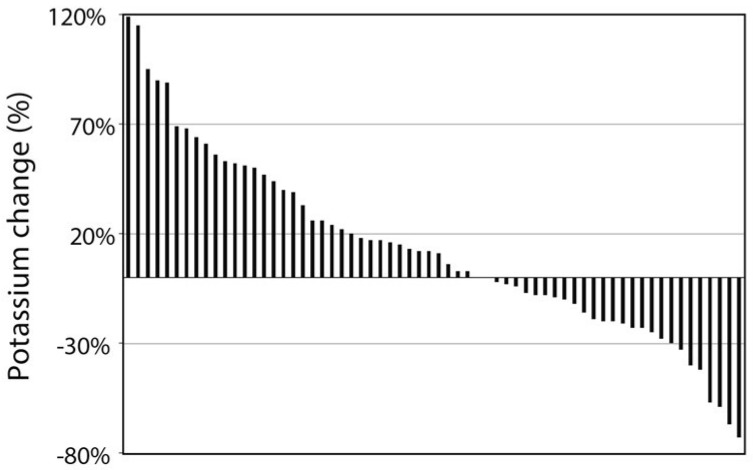
The distribution of the changes in potassium intake in participating subjects after 1 year of intervention. Each line represents % change in potassium intake after 1 year of intervention for an individual participating in the study. Thirty-seven subjects increased and 27 decreased their potassium intake.

**Figure 2:**
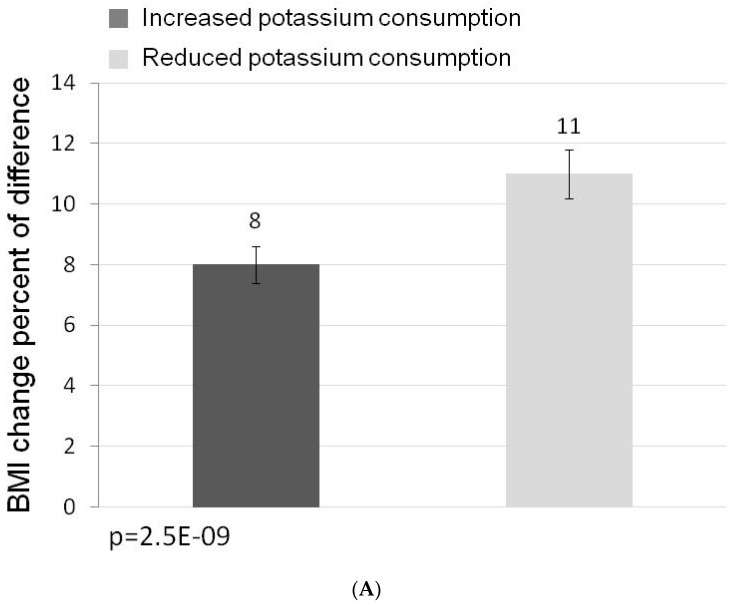

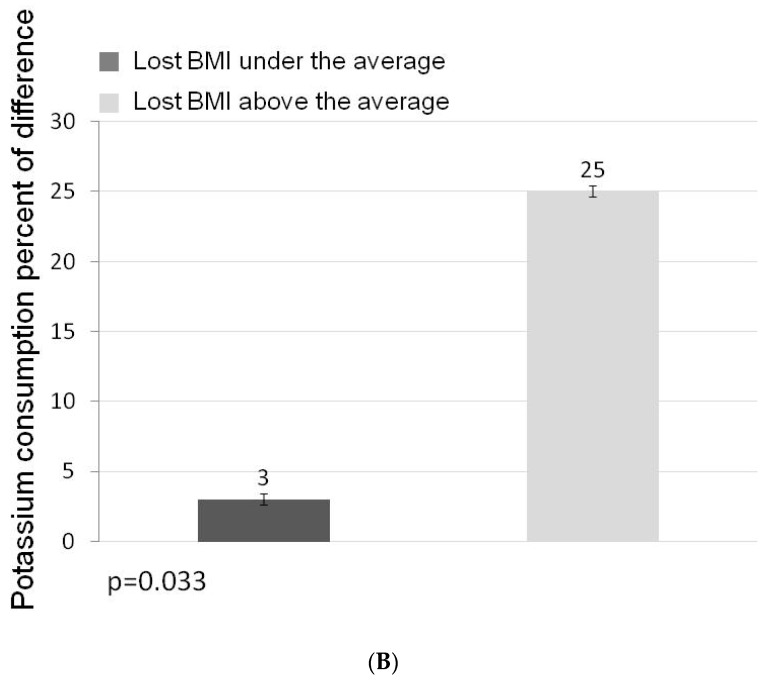
(**A**) Comparison of the reduction in BMI (%) achieved by subjects who increased, vs. those who decreased potassium intake (*n* = 37; 27 respectively). As shown, patients who increased potassium consumption achieved a BMI reduction of 11 ± 0.8%, whereas those who decreased potassium consumption achieved a mean BMI reduction of 8 ± 0.6% (*p* < 0.018). (**B**) The percent change in potassium intake, stratified by BMI loss above or below the average (9.4%). The subgroup showing above the average reduction in BMI (*n* = 29) increased their potassium intake by 25 ± 0.4%, as compared to an increase in potassium consumption of only 3 ± 0.4% in subjects whose achieved reduction in BMI was below the average (*n* = 35; *p* = 0.033).

The distribution of the individual actual percent change in potassium consumption is shown in Figure 1. Since food consumption significantly decreased as a result of the dietary intervention, 27 of 64 the subjects decreased their potassium intake in absolute terms. As shown in Figure 2A, these subjects showed a smaller weight loss than those who, despite lower food consumption, increased dietary potassium (8% vs. 11%; *p* = 0.018). We also divided the study cohort according to the achieved change in BMI and compared the change in potassium consumption in subjects whose achieved reduction in BMI was above the mean for the entire cohort to those who lost less than the mean change in BMI. As shown in Figure 2B, participants in whom the achieved reduction in BMI was higher than the mean, raised their potassium consumption by 25% whereas the subjects whose achieved reduction in BMI was below the mean, raised potassium consumption by only 3% (*p* = 0.033).

To integrate the opposing trends which affected potassium intake, namely, the reduction in overall caloric intake along with the increase in the consumption of potassium-rich food, we calculated “potassium density”, i.e., the potassium consumption per caloric intake. Overall, the potassium density (mg/Kcal/day) increased from 1.3 ± 0.5 to 2.0 ± 0.5 (*p* < 0.000) (Table 2). By the end of the program (1 year), potassium consumption density was 1.9 ± 0.6 mg/Kcal/day in participants whose achieved BMI reduction was less than the mean as compared to 2.1 ± 0.4 (*p* < 0.000) in subjects showing higher than the mean decrease in BMI.

**Figure 3:**
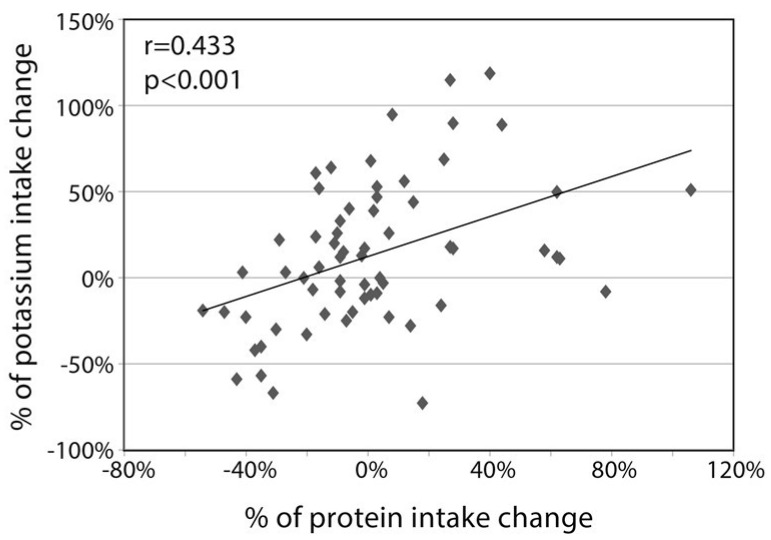
Correlation between the % change in protein consumption and the % change in potassium intake (*n* = 64).

**Figure 4:**
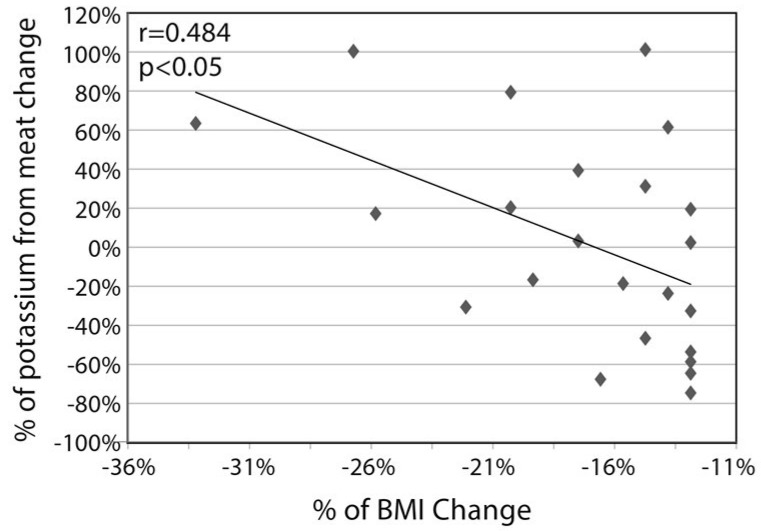
Correlation between the % change in potassium intake from meat products (mostly poultry) and % change in BMI in subjects whose achieved decrease in BMI was above the average (*n* = 23), after 1 year of intervention *p* = 0.019.

Finally, the source of the change in the potassium consumption is of interest. As shown in Figure 3, the % increase in dietary potassium consumption was related to the percent increase in dietary protein consumption (r = 0.433 *p* < 0.001). The change in potassium consumption resulted from mixed changes in the sources in the consumption of various food types. On one hand, there was a sizable decrease in potassium from grains (from 444 ± 313 to 195 ± 129 mg; *p* < 0.001), reflecting the lessening in carbohydrate consumption. In parallel, potassium from vegetables increased from 835 ± 947 to 1277 ± 580 mg; *p* < 0.001), reflecting the change in the prescribed diet composition. There was no significant change in the absolute potassium derived from meat, but in subjects whose achieved decrease in BMI was above the average (*n* = 23), the % change in potassium from meat was negatively related to the **%** change in BMI (r = −0.484, *p* = 0.019; Figure 4).

## 4. Discussion

In this post-hoc analysis of the relation between the change in BMI in the course of an integrative, multidisciplinary lifestyle modification program with concomitant medical treatment of hypertension and hyperlipidemia and changes in nutritional components, we observed that the increase in dietary potassium was a strong predictor of the achieved change in BMI. In fact, in a model that predicted 45% of the variation in the achieved weight loss, a linear stepwise regression analysis revealed that the most influential variable of BMI decline was the increased consumption of dietary potassium. Other nutritional changes that were related to a decline in BMI were an increase in the consumption of caproic acid and a reduction in the consumption of dietary vitamin B6 calcium, total sugars and total energy consumption.

The achieved weight reduction in any intentional weight loss program is a complex outcome of multiple factors including variables such as baseline weight excess, metabolic rate, age, caloric intake, absorptive factors, dietary compliance, physical exercise, level of sedentary life style, co-morbidities (e.g., diabetes, hypogonadism or hyperprolactinemia) and genetic factors. Our results suggest that variation in potassium consumption may also comprise a factor that is related to the achieved weight loss.

There is some evidence from cross-sectional studies that potassium intake may be negatively linked to obesity [13]. In three different reports from Korea and Japan, there appeared to be a trend for lower prevalence of obesity or the MS with higher consumption of potassium [14,15,16]. A recent meta-analysis of epidemiological data in these studies concluded that high potassium intake was associated with a decreased odds ratio for having obesity and the MS [13]. In the Dallas Heart Study, total-body percentage fat was inversely related to the urinary Na/K ratio, which presumably represents the dietary intake of sodium and potassium [17]. In a cross-sectional analysis of a large data base of 24-h diet recalls in US Hispanics-Latinos, Elfassy et al. observed that potassium intake was associated with lower BMI and smaller waist circumference among US-born participants and participants with a longer duration of US residence [18]. In a recent Chinese study, an increase in dietary sodium increased insulin resistance and circulating IL-17A concentrations, and both were significantly attenuated following short-term supplementation of potassium intake [19]. Notably, low dietary potassium was associated with increased risk of incident type 2 diabetes mellitus in African-Americans [20]. Despite these important insights, which collectively suggest a role for dietary potassium in obesity, the metabolic syndrome and type 2 diabetes, we are not aware that potassium intake has been previously linked to the degree of attained weight loss in any former analysis of an interventional trial.

It is notable that the increase in dietary potassium was a stronger predictor of weight loss in this study than such well-established factors as a reduction in sugar consumption and in overall caloric intake. The change in potassium intake during the trial likely reflected two diverse trends: reduction in overall food consumption, which might have had a lowering effect on potassium intake, with a concomitant shift in the type of consumed food, towards potassium-richer products. Hence, whereas overall mean potassium intake did not change, potassium density increased (Table 2). Still, subjects who actually increased their potassium intake had a larger mean decrease in BMI. While of significant potential interest, this new association between the degree of increase in potassium consumption and the size of the reduction in BMI should be viewed with caution. First, post-hoc analyses can generate erroneous observations. Second, obviously neither direct nor indirect causality between dietary potassium and weight loss can be inferred based on our data. Third, the possibility that not potassium per se, but rather a factor linked to the dietary sources of potassium is the actual player in the association between weight loss and dietary potassium should be kept in mind. We analyzed the main food sources from which the increment in dietary potassium appeared to have been derived and increased protein consumption from meat appeared as the largest contributor to the increment in potassium intake. Caproic acid, derived mainly from animal products [21], was also strongly linked to weight loss, perhaps indirectly indicating that meat and milk products consumption may have contributed to the achieved weight loss either through potassium and/or caproic acid intake or through another unidentified components. This interaction also remains to be further verified and investigated. Although it would have been more health-intuitive to attribute the increment in potassium during a transition from a mixture of previous dietary patterns to a healthier diet to higher consumption of potassium-rich fruits and vegetables, data in the present study are inconsistent with this expectation.

The mechanisms through which higher dietary potassium may facilitate weight loss remain elusive. Putative effects might involve a reduction in inflammation and improvement in insulin sensitivity [17], subtle effects on serum potassium which modulate energy balance or neural routes which depend on gut sensing of potassium [22] with beneficial effects on fat deposition/mobilization or energy balance.

## 5. Conclusions 

In conclusion, in a retrospective analysis of the nutritional data base of a study on the effects of an intensive multidisciplinary intervention, combining lifestyle modification and medical treatment of risk factors in subjects with the metabolic syndrome, an increase in dietary potassium consumption emerged as a previously unrecognized, independent and major predictor of the achieved reduction in BMI. Prospective trials in which dietary potassium content is set a priori at low vs. high levels will be needed to determine whether or not an increase in dietary potassium intake can be used to improve weight outcome in the treatment of obesity/metabolic syndrome.

## Figures and Tables

**Table 1:** Clinical, anthropometric and biochemical features of the study’s participants at the initiation of the study and after one year.

Feature (*n*)	Baseline	1 Year	*p* Value ^a^
Age, years (68)	52 ± 12		
Weight, kg (68)	99 ± 17	90 ± 17	<0.000
BMI, kg/m² (68)	35 ± 4	31 ± 4	<0.000
FBM, Kg (62)	40 ± 93	33 ± 8	<0.000
FBM, % (62)	41 ± 1	38.4 ± 1	<0.000
LBM, Kg (62)	56 ± 12	54 ± 12	<0.000
LBM, % (62)	59 ± 11	62 ± 1	<0.000
RMR (43)	1831 ± 404	1759 ± 404	0.128
ADMA, ug/mL (49)	0.57 ± 0.17	0.45 ± 0.10	<0.000
Arginine, ug/mL (49)	42.19 ± 14.17	50.30 ± 19.11	0.025
Systolic Blood Pressure, mmHg (59)	125 ± 11	122 ± 9	0.024
Diastolic Blood Pressure, mmHg (59)	76 ± 9	73 ± 7	0.011
Fasting plasma glucose (mg/dL) (57)	101 ± 15	86 ± 14	<0.000
Total cholesterol (mg/dl) (54)	191 ± 44	172 ± 34	<0.000
Triglycerides (mg/dl) (55)	195 ± 74	134 ± 66	<0.000
HDL (mg/dl) (55)	43 ± 10	47 ± 13	0.001
LDL (mg/dl) (50)	108 ± 33	98 ± 29	0.006
HbA1C (%) (40)	5.9 ± 0.5	5.7 ± 0.4	0.004

a—Paired sample *t*-tests were conducted in order to examine the differences after one year. Abbreviations: BMI, body mass index; FBM, fat body mass; HDL, high density lipoprotein; LBM, lean body mass; LDL, low density lipoprotein; RMR, resting metabolic rate.

**Table 2:** Consumption of selected food components before the dietary intervention and after one year.

Food Components (*n* = 63)	before Treatment	after one Year	*p* Value ^a^
Food Energy (Kcal/day)	2999 ± 1071	1970 ± 641	<0.000
% of carbohydrates (of total calories)	40 ± 11	29 ± 8	<0.000
% of protein (of total calories)	19 ± 5	27 ± 5	<0.000
% of fat (of total calories)	39 ± 7	41 ± 7	<0.030
Potassium (mg/day)	3973 ± 2287	3911 ± 1455	0.752
Potassium density (mg/Kcal/day)	± 1.30.5	0.5 ± 2	<0.000
Sodium (mg/day)	5257 ± 2703	4111 ± 793	<0.000

a—Paired sample t-tests were conducted in order to examine the intake differences after one year Abbreviations: BMI, body mass index; FBM, fat body mass; LBM, lean body mass; RMR, resting metabolic.

**Table 3:** Linear regression model (stepwise) for the prediction of BMI loss in relation to several nutritional features/components and energy consumption.

Step	Predicting Variable	t	β	F change	R^2^ Change	F	R^2^	*p*
1	Caproic acid, % change	−4.010	−0.423 ***	7.412 **	0.119	7.412 **	0.119	<0.000
2	Calcium, % change	2.87	0.335 **	6.005 *	0.082	6.658 **	0.274	0.006
3	Food energy, % change	2.228	0.238 *	4.086 *	0.053	6.305 **	0.327	0.03
4	Potassium, % change	−5.739	−0.865 ***	4.659 *	0.056	6.331 ***	0.383	<0.000
5	Vitamin B6, % of change	3.87	0.542 ***	9.856 **	0.102	7.835 ***	0.485	<0.000
6	Total sugars, % change	2.374	0.239 *	5.634 *	0.055	8.822 ***	0.514	0.022

Notes: Multiple linear regression in a stepwise manner predicting percentage of BMI loss (in Kg/m^2^) by different nutrient intake changes (over 1 year). All macro and micronutrient percentages of intake changes during the study were entered into the model using a stepwise model. Macronutrients included carbohydrates (including dietary fiber and total sugars), proteins and fat (including mono- or poly saturated fatty acids, cholesterol, as well as specific fatty acids such as butyric, caproic, caprylic, capric, lauric, myristic, palmitic, stearic, oleic, linoleic, linolenic, arachidonic, docosahexanoic, palmitoleic, gadoleic, eicosapentaenoic, and erucic). Micronutrients included calcium, iron, magnesium, phosphorus, potassium, sodium, zinc, copper, vitamin a, carotene, vitamin E, vitamin C, thiamin, riboflavin, niacin, vitamin B6, folate, and vitamin B12. The model presented here is the final model including the six variables which were the significant predictors for BMI loss. β is the standardized regression coefficients which is a measure of how strongly the change in the nutrients (and energy) intake influences the BMI loss (the higher the β, the higher the influence). Negative values of β suggest an increase in nutrient intake and a decrease in the BMI, or vice versa. Positive values suggest a reduction in nutrient (and in energy consumption) intake and a decrease in the BMI, or vice versa. In this table, it is shown that the potassium change was the strongest predictor for BMI loss. * *p* < 0.05, ** *p* < 0.01, *** *p* < 0.001.
